# Schisandrin B Induced ROS-Mediated Autophagy and Th1/Th2 Imbalance *via* Selenoproteins in Hepa1-6 Cells

**DOI:** 10.3389/fimmu.2022.857069

**Published:** 2022-03-28

**Authors:** Siran Tan, Zhi Zheng, Tianqi Liu, Xiaoyun Yao, Miao Yu, Yubin Ji

**Affiliations:** ^1^ Engineering Research Center for Medicine, Ministry of Education, Harbin University of Commerce, Harbin, China; ^2^ Jiangxi Province People’s Hospital, First Affiliated Hospital of Nanchang Medical College, Nanchang, China; ^3^ Heilongjiang River Fisheries Research Institute, Chinese Academy of Fishery Sciences, Harbin, China; ^4^ Jiangxi Cancer Hospital, Jiangxi TCM Cancer Center, Nanchang, China

**Keywords:** schisandrin B (Sch B), Hepa1-6, autophagy, oxidative stress, Th1/Th2 imbalance, selenoprotein

## Abstract

Schisandrin B (Sch B) is well-known for its antitumor effect; however, its underlying mechanism remains confusing. Our study aimed to investigate the role of selenoproteins in Sch B-induced autophagy and Th1/Th2 imbalance in Hepa1-6 cells. Hepa1-6 cells were chosen to explore the antitumor mechanism and were treated with 0, 25, 50, and 100 μM of Sch B for 24 h, respectively. We detected the inhibition rate of proliferation, transmission electron microscopy (TEM), monodansylcadaverine (MDC) staining, reactive oxygen species (ROS) level and oxidative stress-related indicators, autophagy-related genes, related Th1/Th2 cytokines, and selenoprotein mRNA expression. Moreover, the heat map, principal component analysis (PCA), and correlation analysis were used for further bioinformatics analysis. The results revealed that Sch B exhibited well-inhibited effects on Hepa1-6 cells. Subsequently, under Sch B treatment, typical autophagy characteristics were increasingly apparent, and the level of punctate MDC staining enhanced and regulated the autophagy-related genes. Overall, Sch B induced autophagy in Hepa1-6 cells. In addition, Sch B-promoted ROS accumulation eventually triggered autophagy initiation. Results of Th1 and Th2 cytokine mRNA expression indicated that Th1/Th2 immune imbalance was observed by Sch B treatment in Hepa1-6 cells. Intriguingly, Sch B downregulated the majority of selenoprotein expression. Also, the heat map results observed significant variation of autophagy-related genes, related Th1/Th2 cytokines, and selenoprotein expression in response to Sch B treatment. PCA outcome suggested the key role of Txnrd1, Txnrd3, Selp, GPX2, Dio3, and Selr with its potential interactions in ROS-mediated autophagy and Th1/Th2 imbalance of Hepa1-6 cells. In conclusion, Sch B induced ROS-mediated autophagy and Th1/Th2 imbalance in Hepa1-6 cells. More importantly, the majority of selenoproteins were intimately involved in the process of autophagy and Th1/Th2 imbalance, Txnrd3, Selp, GPX2, Dio3, and Selr had considerable impacts on the process.

## Introduction

Tumor treatment and uncovering antineoplastic drug mechanisms have attracted intense research efforts. Extensive research has established that natural products extracted and isolated from plants combined with anticancer active substances can help reduce the dosage of anticancer agents and the occurrence of side effects, so these natural active ingredients can be used as cofactors for anticancer treatment (1). Schisandrin B (Sch B), extracted from the traditional Chinese medicinal herb *Schisandra chinensis* Baill., is one of the most active monomers of lignans (2). Existing research recognized the critical role of Sch B in anti-tumor, antioxidant and hepatoprotective effects (3, 4). The antitumor activity of Sch B included promoting apoptosis, inhibiting proliferation, and impairing tumor angiogenesis in various cancer cells (5). There has been little analysis, and systematic understanding of how Sch B exerts its anticancer action is still lacking.

Autophagy is an evolutionarily conserved mechanism for cellular self-digestion that can be involved in maintaining the stability of the internal environment and cellular viability (6). Autophagy is closely linked to tumor suppression and tumor survival. Under normal physiological conditions, autophagy facilitates cells to maintain a self-stable state (7). In case of stress, it can prevent the accumulation of toxic or oncogenic damaged proteins and organelles and inhibits cellular carcinogenesis. Whereas, once tumors are formed, autophagy provides richer nutrients for cancer cells and promotes tumor growth (8). Therefore, autophagy suppresses tumor initiation yet enhances tumor progression (9). Autophagy can be activated under multiple states of stress such as oxidative stress, growth factor deficiency, microbial infection, organelle damage, protein misfolding or aggregation, and DNA damage (10). High levels of reactive oxygen species (ROS) have been detected in the promotion and progression of multiple tumors (11); meanwhile, ROS accumulation is indispensable for the initiation of autophagy (12). Therefore, the mechanisms by which drugs exert anticancer effects by activating oxidative stress and thus inducing autophagy are of notable attention. The ginsenoside Rb1 metabolite K induced both autophagy and apoptosis in HCT-116 cells by producing ROS and activating the c-Jun N-terminal kinase (JNK) pathway (13). Saxifragifolin D increased the expression of *LC3-II, Beclin-1* proteins in MCF-7 and MDA-MB-231 cells, and induced apoptosis and autophagy in breast cancer cells through the ROS-mediated endoplasmic reticulum (ER) stress pathway, therefore inhibiting cancer cell proliferation (14). The induction of ROS and activation of nuclear factor (NF)-κB by gemcitabine (GEM) are required for the effect of antiproliferative synergism in pancreatic cancer cells (15). There is mounting evidence that Th1/Th2 imbalance leads to cancer progression due to their essential role in immunomodulatory function (16, 17). Th1 and Th2 are two subgroups of CD4+ T cells with diverse cytokine production and regulating multiple mechanisms of action in tumor immunity (18, 19). Studies have shown that most tumor tissues are in a state of Th2 cytokine dominance, which is one of the mechanisms of tumor immune escape (20). Compared to healthy subjects, most melanoma patients revealed visibly lower expressions of *interleukin (IL)-2* and *interferon (IFN)-γ* and elevated pathological levels of *IL-4, IL-6,* and *IL-10* demonstrated that the disease was related to Th1/Th2 imbalance (21). Therefore, transferring the Th1/Th2 balance to Th1 to provide a novel protocol for tumor immunotherapy has become a current research hot spot. Saikosaponin A enhanced antitumor immunity by shifting the Th1/Th2 balance toward Th1 and significantly inhibited breast cancer tumor growth and tumor cell proliferation (22). The study aimed to investigate the impact of fire needle stimulation at Sihua acupoints combined with chemotherapy on Th1/Th2 imbalance in non-small cell lung cancer (NSCLC). The results indicated that the expressions of *IL-2* and *IFN-γ* were elevated and of IL-4 and IL-10 were reduced in the treatment group, which illustrated that it can enhance the function of Th1 cells and decrease the function of Th2 cells, altering the imbalance of Th1 and Th2 (23).

Selenoproteins, the catalytic activity center is selenocysteine (Sec), are the main carriers of selenium for physiological functions (24). Sec is mainly located in the redox catalytic site and has a higher redox potential (25). Selenoproteins exist primarily in the form of redox enzymes and carry out very diverse functions such as regulation of cellular oxidative stress, immune function, ER stress, and autophagy, which are integrally related to the development of diverse tumors (26, 27). As mentioned, autophagy is activated under oxidative stress situations. Hence, extensive research suggested selenoproteins as potential molecular targets for anticancer agents that induce oxidative stress, autophagy, and immunity (28–30). Overexpression of Selh in HT22 cells reversed glutamate-induced increase in ROS production and autophagy imbalance (31). The inhibition effect of cyanidin on renal cell carcinoma was correlated with downregulation of early growth response gene 1 (*EGR1*) and *Selw* expression, while modulating the expression of autophagy-related proteins *P62* and *ATG4* (32). Txndr suppression increased the expression of *IL-1β, IL-6, IL-8,* and *IL-10*, which indicated autophagy occurred with a strong immune response in Txn-deficient cardiomyocytes (33).

This study set out to assess the effect of selenoproteins on Sch B inhibiting Hepa1-6 cell proliferation through increasing ROS production and subsequent activation of autophagy and shifting Th1/Th2 balance then regulating immunity. We cultured Hepa1-6 cells with 0-, 25-, 50-, and 100-μM Sch B treatment and then detected the proliferation, autophagy, Th1/Th2 imbalance, and oxidative stress levels in Hepa1-6 cells. Accordingly, to reveal the specific role of selenoproteins in regulating autophagy, oxidative stress, and Th1/Th2 imbalance, we examined the mRNA levels of 25 selenoproteins and performed bioinformatic analysis.

## Materials and Methods

### Cell Culture

Murine hepatocarcinoma cell line Hepa1-6 was gifted by Jiangxi TCM Cancer Center Laboratory. Cells were cultivated in Dulbecco’s modified Eagle’s medium (DMEM) with 10% Fetal Bovine Serum (FBS) and 1% penicillin/streptomycin and maintained at 37°C in a 5% CO_2_ incubator. Hepa1-6 cells incubated with culture medium were added with 25-, 50-, and 100-μM concentrations of Sch B dissolved in dimethyl sulfoxide (DMSO) for 24 h. The same volume of DMSO was substituted as a control.

### Cell Proliferation Assay

The Cell Counting Kit-8 assay (Sant Biotechnology, Shanghai, China) was used to monitor cell proliferation. Here, 5 × 10^3^ cells/well were seeded into 96-well plates and cultivated with Sch B treatment at 0-, 25-, 50-, and 100-μM concentrations for 24 h. Then, 10-μl CCK8 solution was transferred into each well for 2 h, and the absorbance was measured at 450 nm.

### Sections for Electron Microscopy

Hepa1-6 cells were treated with 0-, 25-, 50-, 100-μM concentrations of Sch B dissolved in DMSO for 24 h. The cells were collected into a tube and centrifuged at 250×g for 10 min, then the supernatant was discarded. Cell samples were fixed overnight in 2.5% glutaraldehyde at 4°C, rinsed 3 times with 0.1 M phosphate buffer, and then fixed with osmic acid for 1 h at 4°C. Immediately afterward, they were dehydrated with 50%, 70%, 90%, and 100% ethanol and 100% acetone, respectively, rinsed 3 times with 0.1 M phosphate-buffered saline (pH 7.2), and then fixed with 1% osmic acid for 1 h at 4°C. Afterward, samples were dehydrated with 50%, 70%, 90%, and 100% ethanol and 100% acetone, respectively. Next, the samples were macerated, embedded, aggregated, and then sectioned approximately 50–60 nm with an ultramicrotome. The microphotographs were taken with a transmission electron microscope (GEM-1200ES, Japan).

### Monodansylcadaverine Staining

Monodansylcadaverine (MDC) is a fluorescent chrome that is commonly used to detect specific marker stains for autophagosome formation. The analysis of autophagy was undergone by MDC (Solarbio, Beijing, China). Hepa1-6 cells were treated with 0-, 25-, 50-, 100-μM concentrations of Sch B dissolved in DMSO for 24 h in 12-well plates. Cells were incubated with 0.05 mM MDC in PBS at room temperature away from light for 30 min. The fluorescent images were obtained with a fluorescence microscope with 355-nm excitation filter and 512-nm barrier filter (Leica, Wetzlar, Germany), and cell number was counted to normalize the measurement.

### Detection of Intracellular Reactive Oxygen Species and Oxidative Stress-Related Indicators

Measurement of intracellular ROS used the ROS detection kit (Beyotime, Shanghai, China). After 24-h incubation with 0-, 25-, 50-, 100-μM concentrations of Sch B, cells were incubated with 10 μM 2,7-Dichlorodihydrofluorescein diacetate (DCFH-DA) for 30 min at 37°C. Then, cells were washed with serum-free medium 3 times and measured by fluorescence microscopy (Leica, Wetzlar, Germany). Oxidative stress-related factors superoxide dismutase (SOD), malondialdehyde (MDA), glutathione (GSH), and glutathione peroxidase (GSH-px) were explored according to the protocols of the corresponding kit (Nanjing Jiancheng Bioengineering Institute, China). The absorbance was estimated at 550, 532, 405, and 412 nm, respectively.

### Determination of the mRNA Expression of Autophagy-Related Genes, Related Th1/Th2 Cytokines, and Selenoproteins

Total RNA was extracted from cultured cells by using TRIzol reagent (Invitrogen, Shanghai, China). cDNA was synthesized from 5 mg of the total RNA using oligo dT primers and Superscript II reverse transcriptase according to the manufacturer’s instructions (Promega, Beijing, China). Autophagy-related genes (*LC3, P62, Beclin1, mTOR, ATG1, ATG4, ATG5, ATG7, ATG10, ATG12*), related Th1/Th2 cytokines (*IL-2, TNF-α, IFN-γ, IL-12, IL-4, IL-5, IL-6, IL-10*), and selenoprotein primers (*GPX1, GPX2, GPX3, GPX4, GPX6, Txnrd1, Txnrd2, Txnrd3, Dio1, Dio2, Dio3, Sep15, Selh, Selt, Selw, SPS2, Selm, Selp, Selv, Selo, Selr, Sels, Seli, Selk*) were designed by Primer Premier Software (PREMIER Biosoft International, USA) and are listed in **Table 1**. qRT-PCR was performed to detect the target genes by using Fast Universal SYBR Green Master Mix (Roche, Basel, Switzerland) on the Light Cycler^®^ 480 System (Roche, Basel, Switzerland). Reactions were performed in a 10-μl reaction mixture containing 5 μl of 2× SYBR Green I PCR Master Mix (Roche, Basel, Switzerland), 1 μl of cDNA, 0.2 μl of each primer (10 μM), 0.2 μl of 50× ROX reference Dye II, and 3.4 μl of PCR-grade water. The reaction mixture was then subjected to a thermal profile of denaturation as follows: 1 cycle at 95°C for 30 s and 40 cycles at 95°C for 15 s and 60°C for 30 s. mRNA expression was normalized to β-actin and calculated using the 2^-ΔΔCT^ method.

**Table 1:** The primers used in the present study.

Gene	Forward primer (5′–3′)	Reverse primer (5′–3′)
GPX6	GCCCAGAAGTTGTGGGGTTC	TCCATACTCATAGACGGTGCC
GPX1	AGTCCACCGTGTATCCTTCT	GAGACGCGATTCTCAATGA
GPX2	GCCTCAAGTATGTCCGACCTG	GGAGAACGGGTCATCATAAGGG
GPX3	CCTTTTAAGCAGTATGCAGGCA	CAAGCCAAATGGCCCAAGTT
GPX4	GATGGAGCCCATTCCTGAACC	CCCTGTACTTATCCAGGCAGA
Txnrd 1	CCCACTTGCCCCAACTGTT	GGGAGTGTCTTGGAGGGAC
Txnrd 2	GATCCGGTGGCCTAGCTTG	TCGGGGAGAAGGTTCCACAT
Txnrd 3	GGCAACAGGGTGATGATCTTC	CTGGAAAGTTCGGTCACATCC
Dio1	GCTGAAGCGGCTTGTGATATT	GTTGTCAGGGGCGAATCGG
Dio2	AATTATGCCTCGGAGAAGACCG	GGCAGTTGCCTAGTGAAAGGT
Dio3	CACGGCCTTCATGCTCTGG	CGGTTGTCGTCTGATACGCA
Sep15	CTGGCGACTGCGTTTCAAG	CTGTCCAAGAAGATCGCAAGAG
Selh	TGGACAAGCGCGAGAAACTG	CAGCTCGTACAATGCTCAATGA
Selt	GAGGAGTACATGCGGGTTATCA	CTGACAGGAAAGATGCTATGTGT
Selw	GCCGTTCGAGTCGTGTATTGT	CACTTCAAAGAACCCGGTGAC
SPS2	GCCGGAGTTTCTCCAACTACC	TTCCTGCACCGTCTCTTCCT
Selm	GTTGAATCGCCTAAAGGAGGTG	AGGTCGTCGTGTTCTGAAGC
Selp	CATCTGGTTCAGTGCTTTGATCT	ACCCGTGAGTTATTCCATGAGT
Selv	CTCGTCCTCCAAGACACAAGG	AACTCTAGTGTAGGGTTGGGG
Selo	GCACTGCTACTGTGGACACC	ACTTTGCGACCATCGGCTT
Selr	CTTCGGAGGCGAGGTTTTCC	TCTCAGGGCACTTGGTCACA
Sels	GACCGAGAGCCTGCGATTC	AGCCCTCAGTCGAAGGGAG
Seli	TTGGCTGGCTCCCAATCTTAT	GGTCGAAGTATGTCAGGAGTAGG
Selk	GTTTACATCTCGAATGGTCAGGT	CCCTCTTCCATCGTCGTATCTG
β-actin	CCGCTCTATGAAGGCTACGC	CTCTCGGCTGTGGTGGTGAA
LC3	AGTGAAGTGTAGCAGGATGA	AAGCCTTGTGAACGAGAT
P62	TCCTTCACTCACGCCATGC	CTGCTTGACAGGTATCAGCAC
mTOR	GGACTCTTCCCTGCTGGCTAA	TACGGGTGCCCTGGTTCTG
Beclin1	CGACTGGAGCAGGAAGAAG	TCTGAGCATAACGCATCTGG
ATG1	AAGTTCGAGTTCTCTCGCAAG	CGATGTTTTCGTGCTTTAGTTCC
ATG4	GATGTCAGTGCTCGTCTC	GGAGGATTCTGTGATATTCTTC
ATG5	GGCACCGACCGATTTAGT	GCTGATGGGTTTGCTTTT
ATG7	TCAGATTCAAGCACTTCAGA	GAGGAGATACAACCACAGAG
ATG12	GGTGACGCCAAGAAGAAA	TTGATGAAGTCGCACAGG
IFN-γ	ATGAACGCTACACACTGCATC	CCATCCTTTTGCCAGTTCCTC
TNF-α	CCCTCACACTCAGATCATCTTCT	GCTACGACGTGGGCTACAG
IL-2	TGAGCAGGATGGAGAATTACAGG	GTCCAAGTTCATCTTCTAGGCAC
IL-6	TAGTCCTTCCTACCCCAATTTCC	TTGGTCCTTAGCCACTCCTTC
IL-12	TGGTTTGCCATCGTTTTGCTG	ACAGGTGAGGTTCACTGTTTCT
IL-4	GGTCTCAACCCCCAGCTAGT	GCCGATGATCTCTCTCAAGTGAT
IL-5	CTCTGTTGACAAGCAATGAGACG	TCTTCAGTATGTCTAGCCCCTG
IL-10	GCTCTTACTGACTGGCATGAG	CGCAGCTCTAGGAGCATGTG

### Statistical Analyses

The heat map was generated by https://hiplot.com.cn/basic/heatmap and ranked genes by the degree of expression levels of autophagy-related genes, related Th1/Th2 cytokines, and selenoproteins. SPSS 13.0 program (SPSS, Chicago, IL, USA) was used to execute principal component analysis (PCA), and correlation analysis was performed by Pearson’s correlation coefficient analysis. All data were statistically analyzed by one-way ANOVA, showed a normal distribution, and passed equal variance testing using GraphPad Prism version 8.0 software and SPSS 13.0. The experimental data are expressed as the mean ± SD, and the differences were considered to be significant if *P* < 0.05; * means significantly different (*P* < 0.05) from the control group.

## Results

### Effect of Schisandrin B on the Proliferation of Hepa1-6 Cells

**Figure 1:**
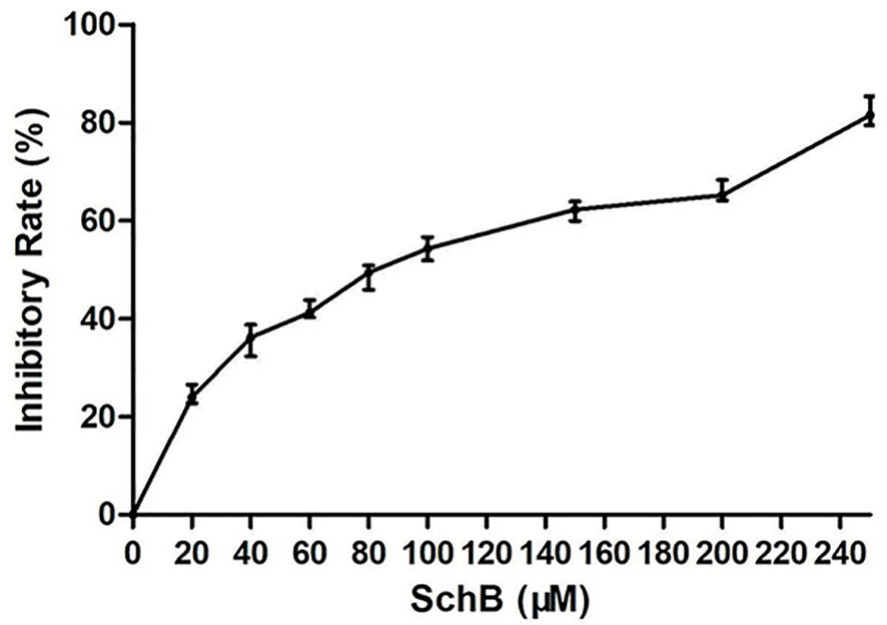
Sch B inhibits proliferation in Hepa1-6 cells. Proliferation curve of Hepa1-6 cells treated with Sch B.

CCK8 proliferation assay aimed to examine the impact on proliferation rates with increased Sch B concentration in Hepa1-6 cells. As can be seen from **Figure 1**, the marked increase in cell inhibition rate was accompanied by increased Sch B treatment.

### Effect of Schisandrin B on Autophagy of Hepa1-6 Cells

**Figure 2:**
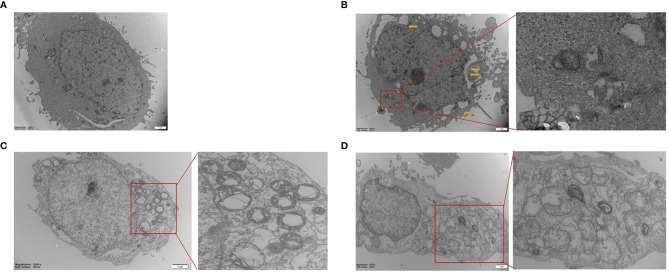
Transmission electron microscope images of Hepa1-6 cells treated with 0 **(A)**, 25-μM **(B)**, 50-μM **(C)**, and 100-μM **(D)** Sch **(B)** The yellow arrows are pointing to the mitochondrial swelling, mitochondrial ridge breakage. An enlarged portion of the spectrogram (red box) is shown on the right showing vacuolar and cytoplasmic localized membrane-bound autophagosome.

Hepa1-6 cells were subjected to transmission electron microscopy (TEM) to reveal Sch B‐induced ultracellular structure. The control group exhibited normal cell morphology (**Figure 2A**). Here, 25-μM Sch B treatment caused cell shrinkage, nuclear condensation, and mitochondrial swelling, and a few autophagosomes were observed (**Figure 2B**). With growing Sch B treatment, typical autophagy characteristics were increasingly apparent, with an elevated number of the autolysosomes and autophagic vacuolization observed in the Hepa1-6 cells (**Figures 2C, D**).

**Figure 3:**
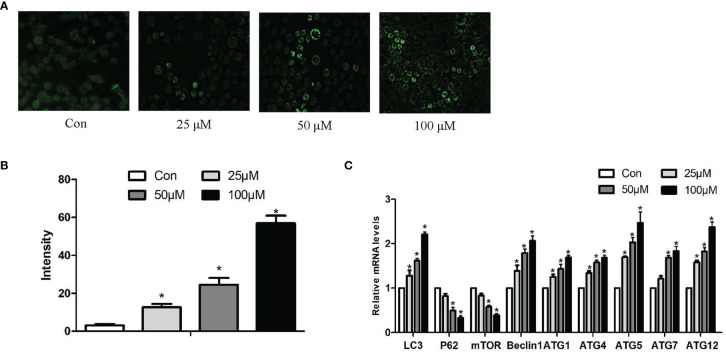
Effects of Sch B on autophagy in Hepa1-6 cells. **(A)** MDC staining was performed in Hepa1-6 cells with Sch B treatment at 0-, 25-, 50-, and 100-μM concentrations. Hepa1-6 cells were visualized using fluorescence microscopy. **(B)** The quantitative analysis of MDC staining. **(C)** Autophagy-related gene mRNA levels in Hepa1-6 cells with 0-, 25-, 50-, and 100-μM Sch B treatment. ∗ shows a significant difference from the corresponding control (*P* < 0.05). n = 3.

The fluorescent dye MDC was used to monitor autophagic vacuoles in Hepa1-6 cells. Normal cells show yellow-green fluorescence; moreover, one hallmark of autophagy is the formation of vacuoles that stain with MDC, resulting in a punctate green fluorescence. As noticed in **Figures 3A, B**, the fluorescence intensity of MDC staining in Hepa1-6 cells was relatively exiguous and weak in the control group. In addition, the level of punctate MDC staining enhanced and outnumbered in a dose-dependent manner with increasing Sch B treatment.

In order to further prove that Sch B induces autophagy of Hepa1-6 cells, we investigated the mRNA expression of autophagy-related genes. qRT-PCR analysis indicated, accompanying increased Sch B concentration, increased levels of LC3, Beclin1, ATG1, ATG4, ATG5, ATG7, ATG10, and ATG12, also, low-level expressions of P62 and mammalian target of rapamycin (mTOR) (**Figure 3C**).

### Effect of Schisandrin B on Oxidative Stress of Hepa1-6 Cells

**Figure 4:**
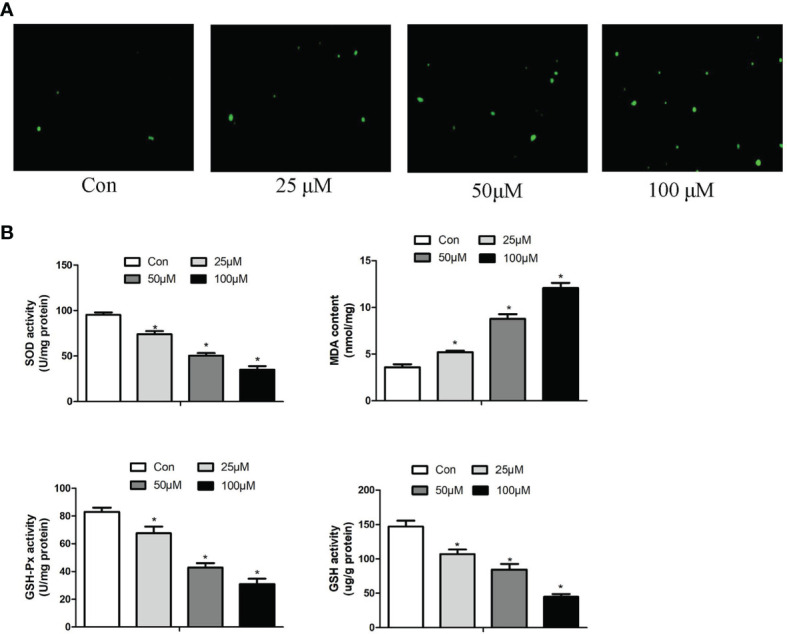
Effects of Sch B on oxidative stress in Hepa1-6 cells. **(A)** ROS generation was performed by immunofluorescence with 0-, 25-, 50-, and 100-μM Sch B treatment in Hepa1-6 cells. Hepa1-6 cells were visualized using fluorescence microscopy. **(B)** Oxidative stress markers of the SOD, MDA, GSH, and GSH-Px contents were measured in Hepa1-6 cells with 0-, 25-, 50-, and 100-μM Sch B treatment. ∗ shows a significant difference from the corresponding control (*P* < 0.05). n = 3.

The production of ROS, the content of MDA, GSH and the activity of GSH-Px, SOD set out to determine whether Sch B induced oxidative stress in Hepa1-6 cells. **Figure 4A** illustrated that the increase in Sch B concentration was related to the ROS activities significantly increased. It can be seen from the data in **Figure 4B** that as the concentration of Sch B rises, there was an observed increase in the levels of MDA and a decline in the content of GSH and the activity of SOD and GSH-Px compared to those of the control group in Hepa1-6 cells.

### Effect of Schisandrin B on Th1/Th2 Imbalance of Hepa1-6 Cells

**Figure 5:**
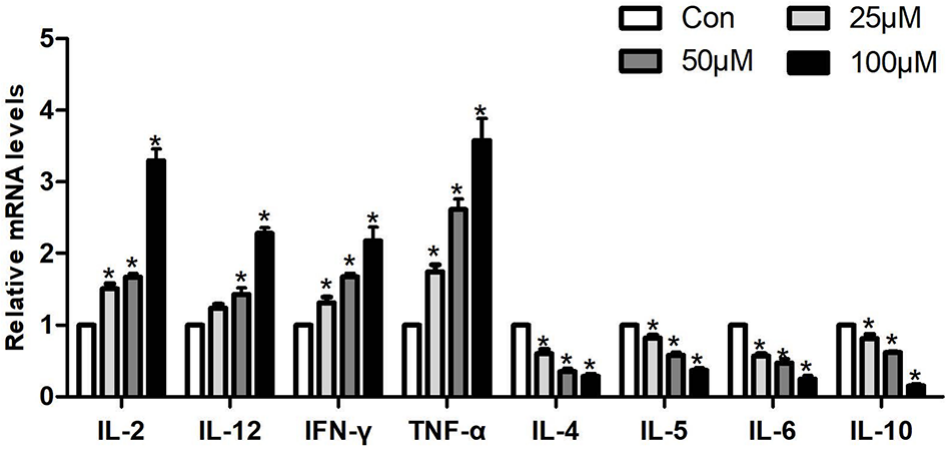
Effects of Sch B on Th1/Th2 imbalance in Hepa1-6 cells. Related Th1/Th2 cytokine mRNA levels with 0-, 25-, 50-, and 100-μM Sch B treatment in Hepa1-6 cells. ∗ shows a significant difference from the corresponding control (*P* < 0.05). n = 3.

In order to explore the effect of Sch B in regulating Th1/Th2 imbalance in Hepa1-6 cells, the mRNA expressions of related Th1/Th2 cytokines were detected. Th1 cytokine contains *IL-2, TNF-α, IFN-γ,* and *IL-12*. Th2 cytokine includes *IL-4, IL-5, IL-6,* and *IL-10*. As revealed in **Figure 5**, Sch B supplement enhanced the expression of *IL-2, TNF-α, IFN-γ,* and *IL-12* and strikingly decreased the expression of *IL-4, IL-5, IL-6,* and *IL-10* (*P* < 0.05). The results indicated the activation of Th1 and the inhibition of Th2 response with Sch B treatment in Hepa1-6 cells. After Sch B treatment, the abnormal expression of cytokines indicated the imbalance of Th1/Th2 occurring in Hepa1-6 cells.

### Effect of Schisandrin B on the Expression of Selenoprotein mRNA Levels of Hepa1-6 Cells

**Figure 6:**
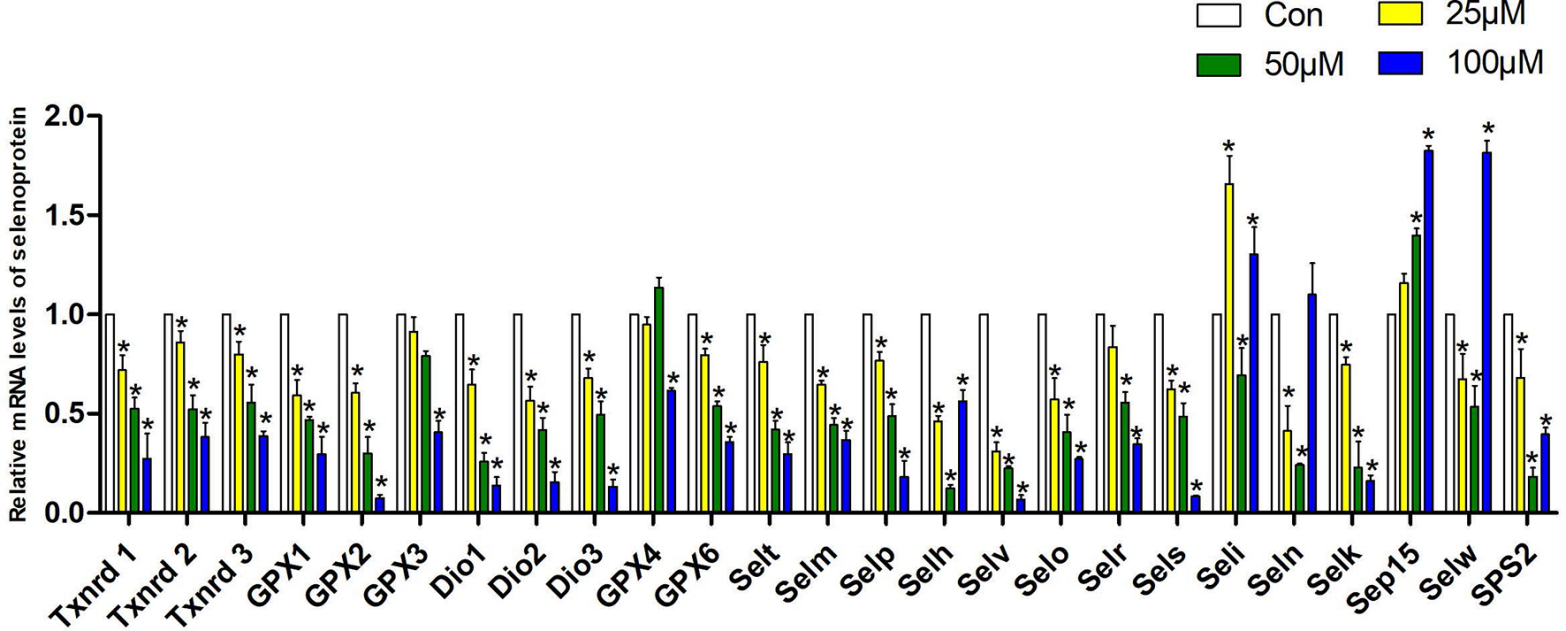
Effects of Sch B on the expression of selenoprotein mRNA levels in Hepa1-6 cells. The 25 selenoprotein mRNA levels with 0-, 25-, 50-, and 100-μM Sch B treatment in Hepa1-6 cells. ∗ shows a significant difference from the corresponding control (*P* < 0.05). n = 3.

We measured the mRNA expression of 25 selenoproteins in Hepa1-6 cells to explore the particular involvement of selenoproteins in Sch B producing oxidative stress hence initiating autophagy and Th1/Th2 imbalance. **Figure 6** presented the results obtained from the expression of 25 selenoproteins on treatment of Sch B at 0, 25, 50, and 100 µM, respectively. *Txnrd1, Txnrd2, Txnrd3, GPX1, GPX2, Dio1, Dio2, Dio3, GPX6, Selt, Selm, Selp, Selv, Selo, Sels, Selk* expression statistically increased with growing Sch B concentration. There is no distinct trend variation of the expression of *GPX3, GPX4, Selh, Seli, Seln, Selw, SPS2,* and *Sep15*.

### Heat Map Analysis

**Figure 7:**
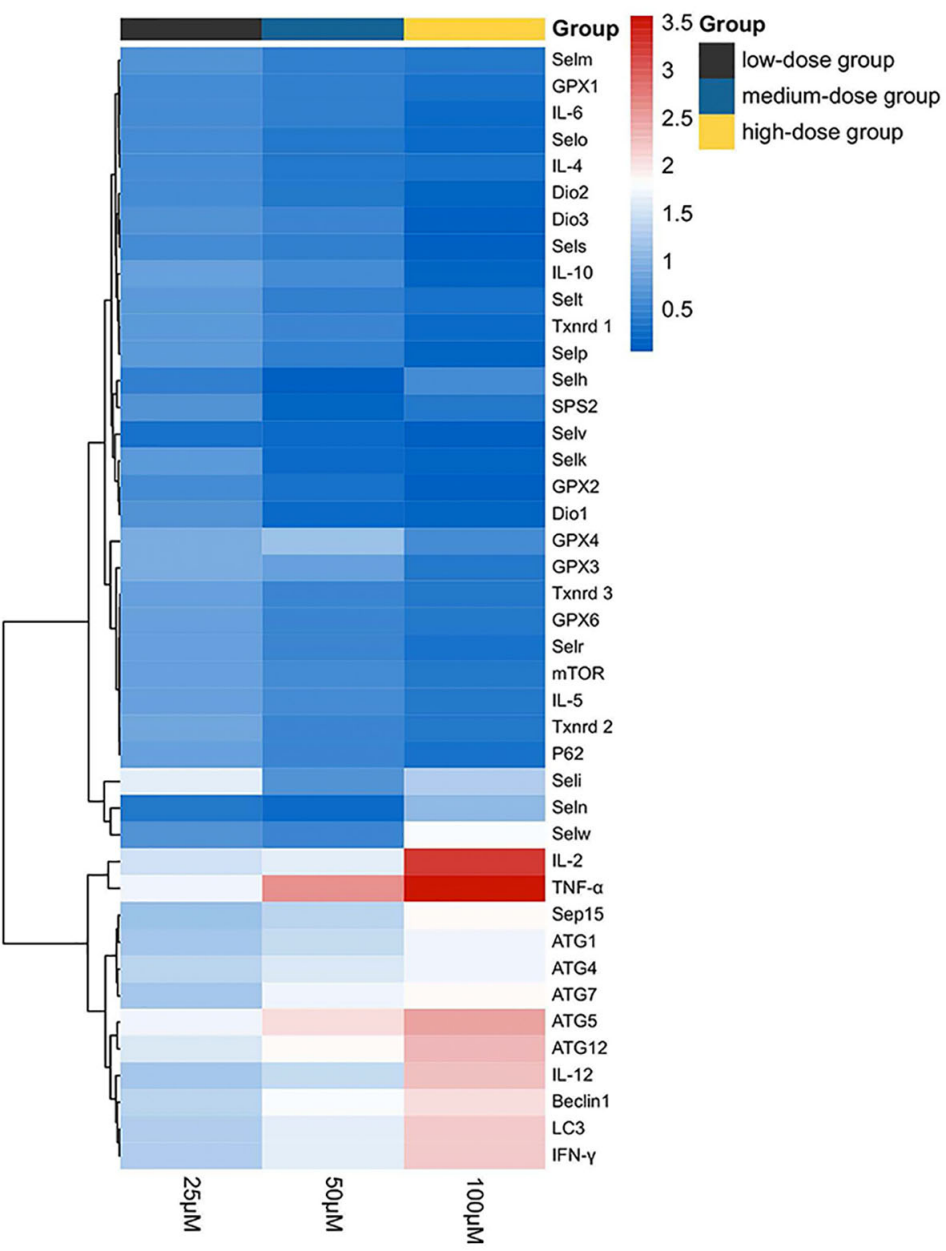
Heat map of autophagy-related genes, related Th1/Th2 cytokines, and selenoprotein mRNA expressions with 25-, 50-, and 100-μM Sch B treatment in Hepa1-6 cells. Rows represent the probe sets. Related genes expressions are shown using the indicated pseudo color scale from blue (0) to red (3.5) relative to values. Red squares represent increased significantly (*P* < 0.05); blue squares represent decreased significantly (*P* < 0.05). Data are presented as mean ± SD.

The results of the heat map analysis were set out in **Figure 7**; selenoproteins, autophagy-related genes, and related Th1/Th2 cytokine expression were shown by blue to red (low to high) coloration within the heat map with the concentration of Sch B (below the heat map) correlating with mRNA expressions. The heat map colored in blue designates the 18 genes, encompassing *Txnrd1, Txnrd2, Txnrd3, GPX1, GPX2, Dio1, Dio2, Dio3, GPX6, Selt, Selm, Selp, Selv, Selo, Sels, Selk, P62, mTOR, IL-4, IL-5, IL-6, IL-10,* had negative levels of correlation between increasing Sch B treatment in Hepa1-6 cells. *LC3, Beclin1, ATG1, ATG4, ATG5, ATG7, ATG10, ATG12, TNF-α, IFN-γ, IL-2, IL-12,* colored in red, had positive level of Sch B growing treatment.

### Principal Component Analysis and Correlation Analysis

**Figure 8:**
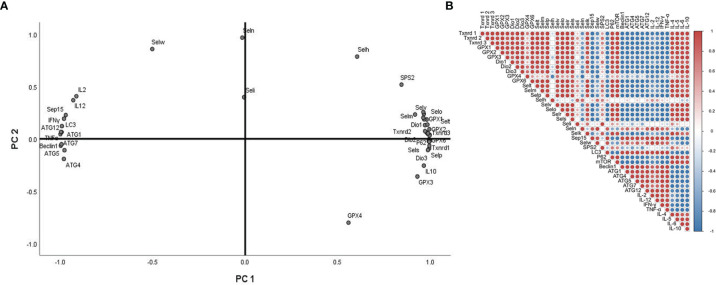
Principal component analysis (PCA) and correlation analysis of autophagy-related genes, related Th1/Th2 cytokines, and selenoprotein mRNA expressions with 0-, 25-, 50-, and 100-μM Sch B treatment in Hepa1-6 cells.

For further factor analysis to explore the particular role of selenoproteins in Sch B inducing autophagy and inflammation in Hepa1-6 cells, the mRNA expression of 25 selenoproteins and autophagy-related genes and related Th1/Th2 cytokines was subjected to PCA (**Figure 8A**) and correlation analysis (**Figure 8B** and **Supplementary Table 2**). PCA revealed 2 major principal components, explaining 85.929% and 10.450% of the variation, respectively. *Txnrd1, Txnrd3, Selp, IL-5, GPX2, Dio3, mTOR,* and *Selr* were highly positively correlated with PC1. *ATG1, Beclin1, TNF-α, ATG5, ATG12,* and *IFN-γ* were highly negatively correlated with PC1. In addition, the correlation analysis exhibited high correlation among selenoproteins with autophagy-related genes and related Th1/Th2 cytokines. Most selenoproteins have a highly positive correlation with *P62, mTOR, IL-4, IL-5, IL-6,* and *IL-10* and a negative correlation with *LC3, Beclin1, ATG1, ATG4, ATG5, ATG7, ATG12, TNF-α, IFN-γ, IL-2,* and *IL-12*.

## Discussion

Prior studies have noted that numerous traditional Chinese medicine and related active compounds have been reported to have potent anticancer properties (34). Sch B is the major anticancer monomer of lignans from the traditional Chinese medicinal herb *S. chinensis* Baill (35). Sch B has been confirmed to inhibit the proliferation of glioma, gastric cancer, and prostate cancer cells *via* induction of apoptosis and autophagy, promotion of antioxidant enzyme release, and inhibition of metastasis and invasion (36). While, some research has been carried out on Sch B, the mechanism by which the antineoplastic effect of Sch B on liver cancer has not been established explicitly. Hence, our data verified that Sch B exerted excellent inhibitory effects against Hepa1-6 cells; also, the suppression was dose-dependent (**Figure 1**). Consequently, to further investigate the mechanism by which Sch B inhibits the proliferation of Hepa1-6 cells, we explored whether Sch B induced oxidative stress that triggered autophagy and the specific role of selenoprotein and its possible biological mechanism.

The induction of autophagy in tumor cells leads to autophagic cell death, suggesting the possibility of utilizing autophagy activation for cancer therapy and providing a fundamentally new direction for the prevention and treatment of tumors (10). In the present study, to investigate whether Sch B induced autophagy in Hepa1-6 cells, three different methods were applied to explore autophagic flux. Firstly, TEM was used to intuitively diagnose morphological hallmarks of autophagy. Autophagosomes are double-membrane-bound vesicles characteristic of autophagy (37). As the concentration of Sch B increases, the typical autophagic ultrastructures were increasingly obvious, the number of autophagosomes was markedly increased in the TEM images (**Figure 2**). Secondly, MDC, an elective marker for autophagosome formation, was used to quantify the induction of autophagy. Normal cells were uniformly stained yellow-green, and autophagosomes were stained with densely packed green granules of varying sizes (38). As illustrated in **Figures 3A, B**, the control group showed weak fluorescence. The increased MDC positive staining strongly suggested that Sch B treatment induced vesicle acidification and rapidly increased the number of autophagosomes, thus enhancing the punctuate MDC staining. The analysis by ImageJ of fluorescence intensity revealed that the autophagy ratio was increased in a dose-dependent manner. Thirdly, we assessed the expression of autophagy-related mRNA expression (**Figure 3C**). *LC3* is the most widely used marker of autophagy, which is involved in the formation of autophagosomes (39). *Beclin-1* plays an essential function in early autophagosome formation by recruiting other autophagy-related proteins through a complex of *Beclin-1* and Vps34 (10). *P62*, a hallmark protein of the autophagic flux downstream, attaches *LC3* and ubiquitinated substrates and is degraded when autophagosome and autolysosome fuse (10). *mTOR* can block autophagy by inhibiting the *ATG1* complex that is involved in the initiation of the autophagy activity (40). Afterward, the ATG-related protein further constructs autophagosomes. The *ATG5-ATG12* covalent protein complex and the *ATG8* coupling are crucial components of the autophagosome membrane (41). *ATG4* mediates the initial lipidation of *LC3* and the cleavage of *LC3* from the autophagosome membrane during the later stages of autophagy (42). In this study, Sch B induced remarkable upregulation of *LC3, Beclin1, ATG1, ATG4, ATG5, ATG7,* and *ATG12* and degradation of *P62* and *mTOR*. These results corroborate the findings of previous work that growing concentration of Sch B induced autophagy; meanwhile, autophagy is one of the causes for the decrease in cell viability induced by Sch B in Hepa1-6 cells.

Evidence of former studies instructed that ROS participated in the regulation of autophagy, which are the switches of cell survival and death (43, 44). Oxidative stress-inducing drugs have preferential anticancer effects, involving the regulation of autophagy. Trichosanthin (TCS) significantly inhibited the growth of human gastric cancer MKN-45 cells by mediating ROS production and NF-κB/p53 pathway (45). Curcumin induced ROS accumulation in cervical cancer cells, thereby inducing apoptosis, autophagy, and cellular senescence, accompanied by upregulation of *p53* and *p21* proteins (46). These previous results confirmed the association between oxidative stress and autophagy. Therefore, here we further investigated whether Sch B overproduced ROS that could be responsible for the initiation of autophagy in Hepa1-6 cells. The production of ROS, the content of MDA and GSH, and the activity of GSH-Px and SOD were examined after treatment with Sch B in Hepa1-6 cells. SOD and GSH-px are both major antioxidant enzymes with efficient ROS-scavenging ability (47). Quantification of MDA is an indicator of lipid peroxidation and ROS-induced damage. GSH is the primary ROS scavenger, and its depletion is considered to contribute to ROS accumulation. In this essay, we investigated that increasing of Sch B concentration promoted ROS accumulation (**Figure 4A**), suppressed SOD activities, elevated the content of MDA, and reduced the activities of GSH and GSH-px (**Figure 4B**). Results demonstrated that increased ROS accumulation induced autophagy and may inhibit Hepa1-6 cell proliferation with increasing of Sch B concentration.

The development and spread of cancer were often accompanied by Th1/Th2 imbalance occurring (48). Th2 drift occurs in a variety of tumors, including non-small cell lung cancer, rectal cancer, ovarian cancer, choriocarcinoma, and melanoma, and it becomes more pronounced as the malignancy of the tumor increases (49). Th1 cells produce *IL-2, TNF-α,* and *IFN-γ* and mediate and participate in cellular immunity and local inflammatory responses. Th2 cells produce *IL-4, IL-5, IL-6,* and *IL-10*, mediate humoral immunity, and are closely associated with the development of hypersensitivity reactions (50). *IL-4* and *IFN-γ* mediate macrophage activation and inhibit *IL-1* and *TNF-α* production. *IL-10 *suppresses Th1 cell proliferation and cytokine production such as *IFN* and *IL-2* and blocks the induction activity of *IL-12. IL-10* has growth factor-like effects on tumor cells, and IFN has obvious cytotoxic effects. *IL-5* synergizes with *IL-2* and *IL-4* to stimulate B-cell growth and differentiation (51). This study revealed that Sch B upregulated the expression of *IL-2, TNF-α, IFN-γ,* and* IL-12* and reduced the expression of *IL-4, IL-5, IL-6,* and *IL-10,* which evidenced that Sch B triggered Th1/Th2 imbalance and shifted Th1/Th2 balance toward Th1 (**Figure 5**).

Selenoproteins were known for their antioxidant roles as ROS scavengers (52). Selenoprotein deficiencies led to excess cellular ROS and reduced antioxidant defense ability, thereby triggering autophagy. Selenoprotein U (Selu) deletion induced autophagy by inhibiting the phosphatidylinositol 3-kinase (PI3K)-Akt-mTOR signaling pathway in rooster Sertoli cells (29). Gpx3 suppression markedly increased ROS levels and promoted autophagy by downregulation of mTOR and increasing the expression of *ATG7, ATG10,* and *ATG12* (53). The supplement of Se could alleviate the imbalance of Th1/Th2 caused by Pb. Meanwhile, the cytokines including *IL-1β, IL-2, IL-8, IL-10,* and *IFN-γ* had a positive correlation with selenoproteins containing *Sepx1, Selo, Selu, Sepp, Sep15, Selw,* and *Selk*. The results indicated that the altered expressions of selenoproteins influenced cytokines related to Th1/Th2 imbalance (54). In chicken dendritic cells, alterations of some selenoprotein expression were correlated with variations of Th1- and Th2-type cytokines such as *IL-12, IFN-γ, *and *IL-10*, thus inducing Th1/Th2 imbalance (55). Accordingly, to explore the specialized role of selenoproteins in ROS-mediated autophagy and Th1/Th2 imbalance in Hepa1-6 cells with growing Sch B treatment, we investigated the mRNA expression of 25 selenoproteins. Based on the results of **Figure 6**, downregulation of the majority of selenoprotein expressions encompassed *Txnrd1, Txnrd2, Txnrd3, GPX1, GPX2, Dio1, Dio2, Dio3, GPX6, Selt, Selm, Selp, Selv, Selo, Sels,* and *Selk*. For in-depth analysis, heat map with clustering analysis of the differentially expressed genes was adopted to assess the expression of 25 selenoproteins with 0-, 25-, 50-, 100-μM Sch B in Hepa1-6 cells. **Figure 7** displayed the heat map results; significant suppression of a spectrum of selenoprotein expression in response to Sch B treatment was observed. Gpx family has prominent antioxidant effects, and its reduced activity resulted in a weakened ROS-scavenging capacity of cells, thus affecting the entire growth phase of cells (56). Txnrd family exerted an irreplaceable antioxidant role, in addition, bound to many proteins and involved in regulating cell growth such as inhibiting apoptosis and autophagy (57, 58). *Sels,* a widely expressed transmembrane protein located in the ER, is critical for ER stress by eliminating misfolding proteins and regulating oxidative stress, apoptosis, and autophagy (59). Together, the former researchers were in agreement with our findings that the majority of selenoprotein expression downregulation arguably is one of the factors most responsible for ROS-mediated autophagy and Th1/Th2 imbalance with Sch B treatment in Hepa1-6 cells. In addition, to identify the principal selenoproteins of Sch B that induced ROS-mediated autophagy and Th1/Th2 imbalance in Hepa1-6 cells, we performed PCA. *Txnrd1, Txnrd3, Selp, IL-5, GPX2, Dio3,* and* Selr *were highly positively correlated with PC1 (**Figure 8A**). Meanwhile, correlation analysis (**Figure 8B**) indicated that autophagy-related genes and related Th1/Th2 cytokines exhibited remarkable correlation with the expression of the majority of selenoproteins including *Txnrd1, Txnrd2, Txnrd3, GPX1, GPX2, GPX3, GPX4, GPX6, Dio1, Dio2, Dio3, Selt, Selm, Selp, Selh, Selv, Selo, Selr,* and *Sels*. The results demonstrated that selenoproteins had essential effects in the regulation of autophagy as well as Th1/Th2 imbalance by Sch B treatment in Hepa1-6 cells. The outcome suggested the key role of these selenoproteins with their potential interactors in ROS-mediated autophagy and Th1/Th2 imbalance of Hepa1-6 cells. Subsequently, we will undertake further in-depth study to investigate the specific mechanism of these selenoproteins in the inhibitory effect of Sch B in Hepa1-6 cells.

## Conclusion

In summary, we found that Sch B treatment triggered the accumulation of ROS and induced the occurrence of cell autophagy, as well as causing Th1/Th2 imbalance and resulting in inhibiting proliferation of Hepa1-6 cells. Meanwhile, selenoproteins exerted irreplaceable roles in regulating autophagy and Th1/Th2 imbalance in Hepa1-6 cells, and *Txnrd1, Txnrd3, Selp, GPX2, Dio3,* and *Selr* had considerable impacts on the process. More importantly, these findings may help us discover selenoproteins with the specific role involved in Sch B’s remarkable antitumor activity and cell death mechanisms, which would be further exploited as a novel small-molecule candidate drug to improve the Sch B antitumor effect.

## Data Availability Statement

The original contributions presented in the study are included in the article/**Supplementary Material**, further inquiries can be directed to the corresponding authors.

## Author Contributions

All authors contributed to the study conception and design. Material preparation and data collection and analysis were performed by ST. The first draft of the article was written by ST. TL, ZZ, and XY carefully examined and revised the article and performed part of the data analysis. All authors read and approved the final article. Article revision was guided by MY and YJ.

## Funding

This project was funded by the Outstanding Young Talents Project of the Central Government’s Reform and Development Fund for Local Universities (Grant No. 2020YQ12), Heilongjiang Provincial Natural Science Foundation of China (Grant No. YQ2020H024), and Supporting Certificate of Heilongjiang Postdoctoral Scientific Research Developmental Fund (Grant No. LBH-Q20027).

## Conflict of Interest

The authors declare that the research was conducted in the absence of any commercial or financial relationships that could be construed as a potential conflict of interest.

## Publisher’s Note

All claims expressed in this article are solely those of the authors and do not necessarily represent those of their affiliated organizations, or those of the publisher, the editors and the reviewers. Any product that may be evaluated in this article, or claim that may be made by its manufacturer, is not guaranteed or endorsed by the publisher.

## Abbreviations

Sch B, schisandrin B; ROS, reactive oxygen species; SOD, superoxide dismutase; MDA, malondialdehyde; GSH, glutathione; GSH-px, glutathione peroxidase; Txnrd1, thioredoxin reductase 1; Txnrd2, thioredoxin reductase 2; Txnrd3, thioredoxin reductase 3; Gpx1, glutathione peroxidase 1; Gpx2, glutathione peroxidase 2; Gpx3, glutathione peroxidase 3; Gpx4, glutathione peroxidase 4; Gpx6, glutathione peroxidase 6; Dio1, deiodinase 1; Dio2, deiodinase 2; Dio3, deiodinase 3; Selt, selenoprotein t; Selw, selenoprotein w; Selk, selenoprotein k; Selm, selenoprotein m; Selh, selenoprotein h; Selu, selenoprotein u; Seli, selenoprotein i; Seln, selenoprotein n; Selo, selenoprotein o; Sels, selenoprotein s; Selp, selenoprotein p; Selp15, 15-kDa selenoprotein; Sps2, selenoprotein synthetase 2; Se, selenium; Sec, selenocysteine; LC3, microtubule-associated protein light chain 3; mTOR, mammalian target of rapamycin; ATG1, autophagy-related gene-1; ATG4, autophagy-related gene-4; ATG5, autophagy-related gene-5; ATG7, autophagy-related gene-7; ATG10, autophagy-related gene-10; TNF-α, tumor necrosis factor α; IL-2, interleukin-2; IL-4, interleukin-4; IL-5, interleukin-5; IL-6, interleukin-6; IL-10, interleukin-10; IL-12, interleukin-12; DMEM, Dulbecco’s modified Eagle’s medium; GAPDH, Glyceraldehyde-3-phosphate Dehydrogenase; qPCR, Quantitative real-time PCR.
