# Supplementary material for: Schisandrin B Induced ROS-Mediated Autophagy and Th1/Th2 Imbalance via Selenoproteins in Hepa1-6 Cells
Source: Front Immunol. 2022 Mar 28;13:857069. doi: 10.3389/fimmu.2022.857069 (PMC8996176; doi:10.3389/fimmu.2022.857069)
Supplement: Supplementary file 14 [file Table_2.docx]

|  | Txnrd1 | Txnrd2 | Txnrd3 | GPX1 | GPX2 | GPX3 | Dio1 | Dio2 | Dio3 | GPX4 | GPX6 | Selt | Selm | Selp | Selh | Selv | Selo | Selr | Sels | Seli | Seln | Selk | Selp15 | Selw | SPS2 | LC3 | P62 | mTOR | Beclin1 | ATG1 | ATG4 | ATG5 | ATG7 | ATG12 | IL2 | IL12 | IFNγ | TNFα | IL4 | IL5 | IL6 | IL10 |
| --- | --- | --- | --- | --- | --- | --- | --- | --- | --- | --- | --- | --- | --- | --- | --- | --- | --- | --- | --- | --- | --- | --- | --- | --- | --- | --- | --- | --- | --- | --- | --- | --- | --- | --- | --- | --- | --- | --- | --- | --- | --- | --- |
| Txnrd1 | 1 | 0.972 | 0.993 | 0.976 | 0.994 | 0.934 | 0.977 | 0.99 | 0.997 | 0.578 | 0.993 | 0.979 | 0.965 | 0.994 | 0.596 | 0.924 | 0.97 | 0.988 | 0.993 | -0.042 | -0.023 | 0.953 | -0.97 | -0.512 | 0.832 | -0.98 | 0.982 | 0.99 | -0.994 | -1 | -0.978 | -0.995 | -0.969 | -0.996 | -0.92 | -0.934 | -0.99 | -0.995 | 0.961 | 0.992 | 0.978 | 0.968 |
| Txnrd2 | 0.972 | 1 | 0.992 | 0.923 | 0.976 | 0.903 | 0.982 | 0.938 | 0.958 | 0.456 | 0.993 | 0.993 | 0.942 | 0.983 | 0.616 | 0.853 | 0.927 | 0.994 | 0.939 | 0.194 | -0.005 | 0.99 | -0.953 | -0.461 | 0.885 | -0.959 | 0.999 | 0.994 | -0.988 | -0.972 | -0.962 | -0.956 | -0.999 | -0.948 | -0.86 | -0.891 | -0.974 | -0.983 | 0.943 | 0.991 | 0.915 | 0.939 |
| Txnrd3 | 0.993 | 0.992 | 1 | 0.961 | 0.994 | 0.918 | 0.989 | 0.974 | 0.984 | 0.51 | 1 | 0.995 | 0.966 | 0.994 | 0.626 | 0.903 | 0.961 | 0.996 | 0.973 | 0.072 | 0.005 | 0.98 | -0.964 | -0.474 | 0.872 | -0.973 | 0.997 | 0.998 | -0.999 | -0.993 | -0.981 | -0.985 | -0.991 | -0.979 | -0.89 | -0.913 | -0.987 | -0.994 | 0.964 | 0.997 | 0.957 | 0.956 |
| GPX1 | 0.976 | 0.923 | 0.961 | 1 | 0.984 | 0.845 | 0.969 | 0.996 | 0.965 | 0.481 | 0.959 | 0.956 | 0.99 | 0.947 | 0.716 | 0.986 | 0.998 | 0.937 | 0.968 | -0.132 | 0.161 | 0.927 | -0.896 | -0.354 | 0.863 | -0.915 | 0.94 | 0.945 | -0.971 | -0.969 | -0.988 | -0.993 | -0.928 | -0.977 | -0.842 | -0.852 | -0.936 | -0.949 | 0.986 | 0.947 | 0.998 | 0.897 |
| GPX2 | 0.994 | 0.976 | 0.994 | 0.984 | 1 | 0.892 | 0.993 | 0.99 | 0.983 | 0.488 | 0.993 | 0.991 | 0.986 | 0.984 | 0.678 | 0.943 | 0.985 | 0.982 | 0.976 | 0.009 | 0.079 | 0.973 | -0.942 | -0.419 | 0.885 | -0.956 | 0.985 | 0.987 | -0.998 | -0.991 | -0.995 | -0.996 | -0.978 | -0.984 | -0.872 | -0.891 | -0.974 | -0.984 | 0.984 | 0.986 | 0.98 | 0.937 |
| GPX3 | 0.934 | 0.903 | 0.918 | 0.845 | 0.892 | 1 | 0.851 | 0.889 | 0.955 | 0.792 | 0.921 | 0.876 | 0.807 | 0.955 | 0.273 | 0.75 | 0.823 | 0.94 | 0.95 | -0.085 | -0.379 | 0.836 | -0.99 | -0.783 | 0.61 | -0.985 | 0.911 | 0.934 | -0.907 | -0.944 | -0.84 | -0.899 | -0.883 | -0.94 | -0.992 | -0.999 | -0.97 | -0.955 | 0.799 | 0.943 | 0.863 | 0.994 |
| Dio1 | 0.977 | 0.982 | 0.989 | 0.969 | 0.993 | 0.851 | 1 | 0.97 | 0.956 | 0.39 | 0.988 | 0.997 | 0.988 | 0.968 | 0.733 | 0.93 | 0.977 | 0.975 | 0.945 | 0.11 | 0.151 | 0.991 | -0.914 | -0.34 | 0.931 | -0.929 | 0.987 | 0.98 | -0.993 | -0.971 | -0.995 | -0.979 | -0.988 | -0.957 | -0.819 | -0.845 | -0.953 | -0.968 | 0.988 | 0.976 | 0.958 | 0.903 |
| Dio2 | 0.99 | 0.938 | 0.974 | 0.996 | 0.99 | 0.889 | 0.97 | 1 | 0.984 | 0.546 | 0.972 | 0.962 | 0.98 | 0.969 | 0.656 | 0.968 | 0.99 | 0.957 | 0.987 | -0.132 | 0.073 | 0.931 | -0.931 | -0.435 | 0.837 | -0.946 | 0.954 | 0.963 | -0.98 | -0.986 | -0.984 | -0.999 | -0.939 | -0.992 | -0.887 | -0.896 | -0.962 | -0.97 | 0.976 | 0.966 | 0.998 | 0.933 |
| Dio3 | 0.997 | 0.958 | 0.984 | 0.965 | 0.983 | 0.955 | 0.956 | 0.984 | 1 | 0.641 | 0.984 | 0.962 | 0.944 | 0.993 | 0.536 | 0.909 | 0.954 | 0.982 | 0.998 | -0.092 | -0.091 | 0.929 | -0.98 | -0.573 | 0.785 | -0.988 | 0.97 | 0.983 | -0.983 | -0.998 | -0.959 | -0.988 | -0.951 | -0.998 | -0.947 | -0.957 | -0.993 | -0.993 | 0.938 | 0.988 | 0.972 | 0.982 |
| GPX4 | 0.578 | 0.456 | 0.51 | 0.481 | 0.488 | 0.792 | 0.39 | 0.546 | 0.641 | 1 | 0.514 | 0.419 | 0.371 | 0.595 | -0.262 | 0.397 | 0.428 | 0.544 | 0.662 | -0.523 | -0.726 | 0.337 | -0.704 | -0.941 | 0.028 | -0.687 | 0.478 | 0.534 | -0.496 | -0.598 | -0.405 | -0.537 | -0.418 | -0.629 | -0.845 | -0.81 | -0.638 | -0.596 | 0.352 | 0.559 | 0.532 | 0.734 |
| GPX6 | 0.993 | 0.993 | 1 | 0.959 | 0.993 | 0.921 | 0.988 | 0.972 | 0.984 | 0.514 | 1 | 0.994 | 0.963 | 0.995 | 0.619 | 0.899 | 0.958 | 0.997 | 0.973 | 0.075 | -0.004 | 0.98 | -0.966 | -0.481 | 0.869 | -0.974 | 0.997 | 0.999 | -0.999 | -0.992 | -0.979 | -0.983 | -0.991 | -0.979 | -0.893 | -0.916 | -0.988 | -0.995 | 0.962 | 0.998 | 0.955 | 0.958 |
| Selt | 0.979 | 0.993 | 0.995 | 0.956 | 0.991 | 0.876 | 0.997 | 0.962 | 0.962 | 0.419 | 0.994 | 1 | 0.974 | 0.979 | 0.69 | 0.904 | 0.962 | 0.987 | 0.947 | 0.141 | 0.089 | 0.995 | -0.934 | -0.39 | 0.917 | -0.945 | 0.996 | 0.99 | -0.995 | -0.976 | -0.987 | -0.975 | -0.996 | -0.958 | -0.839 | -0.868 | -0.966 | -0.979 | 0.975 | 0.986 | 0.946 | 0.922 |
| Selm | 0.965 | 0.942 | 0.966 | 0.99 | 0.986 | 0.807 | 0.988 | 0.98 | 0.944 | 0.371 | 0.963 | 0.974 | 1 | 0.94 | 0.785 | 0.975 | 0.996 | 0.94 | 0.94 | -0.003 | 0.24 | 0.96 | -0.874 | -0.27 | 0.926 | -0.894 | 0.953 | 0.948 | -0.975 | -0.957 | -0.998 | -0.982 | -0.951 | -0.954 | -0.789 | -0.809 | -0.923 | -0.941 | 1 | 0.946 | 0.979 | 0.868 |
| Selp | 0.994 | 0.983 | 0.994 | 0.947 | 0.984 | 0.955 | 0.968 | 0.969 | 0.993 | 0.595 | 0.995 | 0.979 | 0.94 | 1 | 0.543 | 0.88 | 0.941 | 0.997 | 0.984 | 0.025 | -0.096 | 0.957 | -0.986 | -0.564 | 0.815 | -0.992 | 0.99 | 0.997 | -0.991 | -0.996 | -0.96 | -0.979 | -0.977 | -0.986 | -0.933 | -0.951 | -0.998 | -1 | 0.937 | 0.999 | 0.95 | 0.982 |
| Selh | 0.596 | 0.616 | 0.626 | 0.716 | 0.678 | 0.273 | 0.733 | 0.656 | 0.536 | -0.262 | 0.619 | 0.69 | 0.785 | 0.543 | 1 | 0.78 | 0.754 | 0.566 | 0.528 | 0.189 | 0.783 | 0.717 | -0.397 | 0.384 | 0.901 | -0.433 | 0.621 | 0.584 | -0.65 | -0.573 | -0.75 | -0.655 | -0.653 | -0.564 | -0.239 | -0.271 | -0.497 | -0.544 | 0.795 | 0.569 | 0.675 | 0.379 |
| Selv | 0.924 | 0.853 | 0.903 | 0.986 | 0.943 | 0.75 | 0.93 | 0.968 | 0.909 | 0.397 | 0.899 | 0.904 | 0.975 | 0.88 | 0.78 | 1 | 0.986 | 0.866 | 0.919 | -0.207 | 0.292 | 0.873 | -0.809 | -0.226 | 0.854 | -0.835 | 0.874 | 0.877 | -0.919 | -0.914 | -0.961 | -0.958 | -0.864 | -0.93 | -0.758 | -0.763 | -0.864 | -0.881 | 0.971 | 0.879 | 0.981 | 0.814 |
| Selo | 0.97 | 0.927 | 0.961 | 0.998 | 0.985 | 0.823 | 0.977 | 0.99 | 0.954 | 0.428 | 0.958 | 0.962 | 0.996 | 0.941 | 0.754 | 0.986 | 1 | 0.935 | 0.955 | -0.087 | 0.208 | 0.939 | -0.881 | -0.307 | 0.892 | -0.902 | 0.942 | 0.943 | -0.971 | -0.962 | -0.993 | -0.989 | -0.935 | -0.966 | -0.815 | -0.828 | -0.927 | -0.942 | 0.994 | 0.943 | 0.992 | 0.88 |
| Selr | 0.988 | 0.994 | 0.996 | 0.937 | 0.982 | 0.94 | 0.975 | 0.957 | 0.982 | 0.544 | 0.997 | 0.987 | 0.94 | 0.997 | 0.566 | 0.866 | 0.935 | 1 | 0.968 | 0.1 | -0.069 | 0.973 | -0.978 | -0.532 | 0.843 | -0.983 | 0.997 | 1 | -0.992 | -0.989 | -0.962 | -0.971 | -0.99 | -0.972 | -0.909 | -0.932 | -0.993 | -0.997 | 0.939 | 0.999 | 0.935 | 0.969 |
| Sels | 0.993 | 0.939 | 0.973 | 0.968 | 0.976 | 0.95 | 0.945 | 0.987 | 0.998 | 0.662 | 0.973 | 0.947 | 0.94 | 0.984 | 0.528 | 0.919 | 0.955 | 0.968 | 1 | -0.152 | -0.09 | 0.909 | -0.972 | -0.575 | 0.764 | -0.981 | 0.954 | 0.97 | -0.974 | -0.994 | -0.953 | -0.987 | -0.933 | -0.999 | -0.95 | -0.956 | -0.986 | -0.985 | 0.933 | 0.976 | 0.978 | 0.977 |
| Seli | -0.042 | 0.194 | 0.072 | -0.132 | 0.009 | -0.085 | 0.11 | -0.132 | -0.092 | -0.523 | 0.075 | 0.141 | -0.003 | 0.025 | 0.189 | -0.207 | -0.087 | 0.1 | -0.152 | 1 | 0.149 | 0.24 | 0.014 | 0.24 | 0.323 | 0.026 | 0.147 | 0.092 | -0.055 | 0.043 | -0.024 | 0.083 | -0.206 | 0.129 | 0.208 | 0.137 | 0.002 | -0.021 | 0.017 | 0.065 | -0.18 | -0.068 |
| Seln | -0.023 | -0.005 | 0.005 | 0.161 | 0.079 | -0.379 | 0.151 | 0.073 | -0.091 | -0.726 | -0.004 | 0.089 | 0.24 | -0.096 | 0.783 | 0.292 | 0.208 | -0.069 | -0.09 | 0.149 | 1 | 0.133 | 0.258 | 0.866 | 0.453 | 0.217 | -0.002 | -0.047 | -0.038 | 0.053 | -0.183 | -0.061 | -0.043 | 0.051 | 0.396 | 0.375 | 0.148 | 0.094 | 0.254 | -0.066 | 0.113 | -0.272 |
| Selk | 0.953 | 0.99 | 0.98 | 0.927 | 0.973 | 0.836 | 0.991 | 0.931 | 0.929 | 0.337 | 0.98 | 0.995 | 0.96 | 0.957 | 0.717 | 0.873 | 0.939 | 0.973 | 0.909 | 0.24 | 0.133 | 1 | -0.904 | -0.331 | 0.942 | -0.915 | 0.988 | 0.976 | -0.98 | -0.949 | -0.973 | -0.947 | -0.996 | -0.923 | -0.788 | -0.824 | -0.94 | -0.956 | 0.963 | 0.969 | 0.911 | 0.887 |
| Slep15 | -0.97 | -0.953 | -0.964 | -0.896 | -0.942 | -0.99 | -0.914 | -0.931 | -0.98 | -0.704 | -0.966 | -0.934 | -0.874 | -0.986 | -0.397 | -0.809 | -0.881 | -0.978 | -0.972 | 0.014 | 0.258 | -0.904 | 1 | 0.692 | -0.714 | 0.999 | -0.959 | -0.975 | 0.955 | 0.977 | 0.902 | 0.942 | 0.939 | 0.967 | 0.973 | 0.987 | 0.994 | 0.986 | -0.868 | -0.98 | -0.906 | -0.999 |
| Selw | -0.512 | -0.461 | -0.474 | -0.354 | -0.419 | -0.783 | -0.34 | -0.435 | -0.573 | -0.941 | -0.481 | -0.39 | -0.27 | -0.564 | 0.384 | -0.226 | -0.307 | -0.532 | -0.575 | 0.24 | 0.866 | -0.331 | 0.692 | 1 | 0.005 | 0.664 | -0.467 | -0.515 | 0.449 | 0.537 | 0.322 | 0.443 | 0.418 | 0.542 | 0.802 | 0.785 | 0.608 | 0.563 | -0.255 | -0.534 | -0.4 | -0.708 |
| SPS2 | 0.832 | 0.885 | 0.872 | 0.863 | 0.885 | 0.61 | 0.931 | 0.837 | 0.785 | 0.028 | 0.869 | 0.917 | 0.926 | 0.815 | 0.901 | 0.854 | 0.892 | 0.843 | 0.764 | 0.323 | 0.453 | 0.942 | -0.714 | 0.005 | 1 | -0.736 | 0.881 | 0.852 | -0.882 | -0.818 | -0.921 | -0.851 | -0.906 | -0.791 | -0.554 | -0.597 | -0.782 | -0.815 | 0.935 | 0.839 | 0.83 | 0.69 |
| LC3 | -0.98 | -0.959 | -0.973 | -0.915 | -0.956 | -0.985 | -0.929 | -0.946 | -0.988 | -0.687 | -0.974 | -0.945 | -0.894 | -0.992 | -0.433 | -0.835 | -0.902 | -0.983 | -0.981 | 0.026 | 0.217 | -0.915 | 0.999 | 0.664 | -0.736 | 1 | -0.966 | -0.981 | 0.966 | 0.985 | 0.92 | 0.956 | 0.947 | 0.978 | 0.969 | 0.982 | 0.997 | 0.992 | -0.888 | -0.986 | -0.924 | -0.998 |
| P62 | 0.982 | 0.999 | 0.997 | 0.94 | 0.985 | 0.911 | 0.987 | 0.954 | 0.97 | 0.478 | 0.997 | 0.996 | 0.953 | 0.99 | 0.621 | 0.874 | 0.942 | 0.997 | 0.954 | 0.147 | -0.002 | 0.988 | -0.959 | -0.467 | 0.881 | -0.966 | 1 | 0.998 | -0.994 | -0.982 | -0.971 | -0.969 | -0.998 | -0.962 | -0.874 | -0.901 | -0.981 | -0.989 | 0.953 | 0.995 | 0.933 | 0.947 |
| mTOR | 0.99 | 0.994 | 0.998 | 0.945 | 0.987 | 0.934 | 0.98 | 0.963 | 0.983 | 0.534 | 0.999 | 0.99 | 0.948 | 0.997 | 0.584 | 0.877 | 0.943 | 1 | 0.97 | 0.092 | -0.047 | 0.976 | -0.975 | -0.515 | 0.852 | -0.981 | 0.998 | 1 | -0.995 | -0.991 | -0.968 | -0.975 | -0.991 | -0.975 | -0.904 | -0.927 | -0.992 | -0.997 | 0.947 | 1 | 0.942 | 0.966 |
| Beclin1 | -0.994 | -0.988 | -0.999 | -0.971 | -0.998 | -0.907 | -0.993 | -0.98 | -0.983 | -0.496 | -0.999 | -0.995 | -0.975 | -0.991 | -0.65 | -0.919 | -0.971 | -0.992 | -0.974 | -0.055 | -0.038 | -0.98 | 0.955 | 0.449 | -0.882 | 0.966 | -0.994 | -0.995 | 1 | 0.992 | 0.988 | 0.99 | 0.988 | 0.981 | 0.881 | 0.903 | 0.982 | 0.991 | -0.974 | -0.994 | -0.966 | -0.948 |
| ATG1 | -1 | -0.972 | -0.993 | -0.969 | -0.991 | -0.944 | -0.971 | -0.986 | -0.998 | -0.598 | -0.992 | -0.976 | -0.957 | -0.996 | -0.573 | -0.914 | -0.962 | -0.989 | -0.994 | 0.043 | 0.053 | -0.949 | 0.977 | 0.537 | -0.818 | 0.985 | -0.982 | -0.991 | 0.992 | 1 | 0.972 | 0.992 | 0.967 | 0.996 | 0.93 | 0.944 | 0.994 | 0.997 | -0.952 | -0.994 | -0.973 | -0.975 |
| ATG4 | -0.978 | -0.962 | -0.981 | -0.988 | -0.995 | -0.84 | -0.995 | -0.984 | -0.959 | -0.405 | -0.979 | -0.987 | -0.998 | -0.96 | -0.75 | -0.961 | -0.993 | -0.962 | -0.953 | -0.024 | -0.183 | -0.973 | 0.902 | 0.322 | -0.921 | 0.92 | -0.971 | -0.968 | 0.988 | 0.972 | 1 | 0.989 | 0.969 | 0.966 | 0.819 | 0.839 | 0.945 | 0.961 | -0.997 | -0.966 | -0.978 | -0.896 |
| ATG5 | -0.995 | -0.956 | -0.985 | -0.993 | -0.996 | -0.899 | -0.979 | -0.999 | -0.988 | -0.537 | -0.983 | -0.975 | -0.982 | -0.979 | -0.655 | -0.958 | -0.989 | -0.971 | -0.987 | 0.083 | -0.061 | -0.947 | 0.942 | 0.443 | -0.851 | 0.956 | -0.969 | -0.975 | 0.99 | 0.992 | 0.989 | 1 | 0.956 | 0.993 | 0.89 | 0.902 | 0.972 | 0.98 | -0.978 | -0.978 | -0.993 | -0.942 |
| ATG7 | -0.969 | -0.999 | -0.991 | -0.928 | -0.978 | -0.883 | -0.988 | -0.939 | -0.951 | -0.418 | -0.991 | -0.996 | -0.951 | -0.977 | -0.653 | -0.864 | -0.935 | -0.99 | -0.933 | -0.206 | -0.043 | -0.996 | 0.939 | 0.418 | -0.906 | 0.947 | -0.998 | -0.991 | 0.988 | 0.967 | 0.969 | 0.956 | 1 | 0.943 | 0.839 | 0.871 | 0.965 | 0.977 | -0.953 | -0.986 | -0.917 | -0.924 |
| ATG12 | -0.996 | -0.948 | -0.979 | -0.977 | -0.984 | -0.94 | -0.957 | -0.992 | -0.998 | -0.629 | -0.979 | -0.958 | -0.954 | -0.986 | -0.564 | -0.93 | -0.966 | -0.972 | -0.999 | 0.129 | 0.051 | -0.923 | 0.967 | 0.542 | -0.791 | 0.978 | -0.962 | -0.975 | 0.981 | 0.996 | 0.966 | 0.993 | 0.943 | 1 | 0.936 | 0.944 | 0.985 | 0.987 | -0.948 | -0.98 | -0.984 | -0.97 |
| IL2 | -0.92 | -0.86 | -0.89 | -0.842 | -0.872 | -0.992 | -0.819 | -0.887 | -0.947 | -0.845 | -0.893 | -0.839 | -0.789 | -0.933 | -0.239 | -0.758 | -0.815 | -0.909 | -0.95 | 0.208 | 0.396 | -0.788 | 0.973 | 0.802 | -0.554 | 0.969 | -0.874 | -0.904 | 0.881 | 0.93 | 0.819 | 0.89 | 0.839 | 0.936 | 1 | 0.997 | 0.951 | 0.933 | -0.778 | -0.916 | -0.867 | -0.983 |
| IL12 | -0.934 | -0.891 | -0.913 | -0.852 | -0.891 | -0.999 | -0.845 | -0.896 | -0.957 | -0.81 | -0.916 | -0.868 | -0.809 | -0.951 | -0.271 | -0.763 | -0.828 | -0.932 | -0.956 | 0.137 | 0.375 | -0.824 | 0.987 | 0.785 | -0.597 | 0.982 | -0.901 | -0.927 | 0.903 | 0.944 | 0.839 | 0.902 | 0.871 | 0.944 | 0.997 | 1 | 0.967 | 0.951 | -0.799 | -0.937 | -0.873 | -0.993 |
| IFNγ | -0.99 | -0.974 | -0.987 | -0.936 | -0.974 | -0.97 | -0.953 | -0.962 | -0.993 | -0.638 | -0.988 | -0.966 | -0.923 | -0.998 | -0.497 | -0.864 | -0.927 | -0.993 | -0.986 | 0.002 | 0.148 | -0.94 | 0.994 | 0.608 | -0.782 | 0.997 | -0.981 | -0.992 | 0.982 | 0.994 | 0.945 | 0.972 | 0.965 | 0.985 | 0.951 | 0.967 | 1 | 0.998 | -0.918 | -0.995 | -0.942 | -0.991 |
| TNFα | -0.995 | -0.983 | -0.994 | -0.949 | -0.984 | -0.955 | -0.968 | -0.97 | -0.993 | -0.596 | -0.995 | -0.979 | -0.941 | -1 | -0.544 | -0.881 | -0.942 | -0.997 | -0.985 | -0.021 | 0.094 | -0.956 | 0.986 | 0.563 | -0.815 | 0.992 | -0.989 | -0.997 | 0.991 | 0.997 | 0.961 | 0.98 | 0.977 | 0.987 | 0.933 | 0.951 | 0.998 | 1 | -0.937 | -0.999 | -0.951 | -0.982 |
| IL4 | 0.961 | 0.943 | 0.964 | 0.986 | 0.984 | 0.799 | 0.988 | 0.976 | 0.938 | 0.352 | 0.962 | 0.975 | 1 | 0.937 | 0.795 | 0.971 | 0.994 | 0.939 | 0.933 | 0.017 | 0.254 | 0.963 | -0.868 | -0.255 | 0.935 | -0.888 | 0.953 | 0.947 | -0.974 | -0.952 | -0.997 | -0.978 | -0.953 | -0.948 | -0.778 | -0.799 | -0.918 | -0.937 | 1 | 0.944 | 0.974 | 0.861 |
| IL5 | 0.992 | 0.991 | 0.997 | 0.947 | 0.986 | 0.943 | 0.976 | 0.966 | 0.988 | 0.559 | 0.998 | 0.986 | 0.946 | 0.999 | 0.569 | 0.879 | 0.943 | 0.999 | 0.976 | 0.065 | -0.066 | 0.969 | -0.98 | -0.534 | 0.839 | -0.986 | 0.995 | 1 | -0.994 | -0.994 | -0.966 | -0.978 | -0.986 | -0.98 | -0.916 | -0.937 | -0.995 | -0.999 | 0.944 | 1 | 0.946 | 0.973 |
| IL6 | 0.978 | 0.915 | 0.957 | 0.998 | 0.98 | 0.863 | 0.958 | 0.998 | 0.972 | 0.532 | 0.955 | 0.946 | 0.979 | 0.95 | 0.675 | 0.981 | 0.992 | 0.935 | 0.978 | -0.18 | 0.113 | 0.911 | -0.906 | -0.4 | 0.83 | -0.924 | 0.933 | 0.942 | -0.966 | -0.973 | -0.978 | -0.993 | -0.917 | -0.984 | -0.867 | -0.873 | -0.942 | -0.951 | 0.974 | 0.946 | 1 | 0.911 |
| IL10 | 0.968 | 0.939 | 0.956 | 0.897 | 0.937 | 0.994 | 0.903 | 0.933 | 0.982 | 0.734 | 0.958 | 0.922 | 0.868 | 0.982 | 0.379 | 0.814 | 0.88 | 0.969 | 0.977 | -0.068 | -0.272 | 0.887 | -0.999 | -0.708 | 0.69 | -0.998 | 0.947 | 0.966 | -0.948 | -0.975 | -0.896 | -0.942 | -0.924 | -0.97 | -0.983 | -0.993 | -0.991 | -0.982 | 0.861 | 0.973 | 0.911 | 1 |

Table 2 Correlation analysis of Pearson correlation coefficients of selenoproteins, autophagy-related and related Th1/Th2 cytokines in hapa1-6 cells treated with MB.
